# Primary healthcare system capacities for responding to storm and flood-related health problems: a case study from a rural district in central Vietnam

**DOI:** 10.3402/gha.v7.23007

**Published:** 2014-12-08

**Authors:** Hoang Van Minh, Tran Tuan Anh, Joacim Rocklöv, Kim Bao Giang, Le Quynh Trang, Klas-Göran Sahlen, Maria Nilsson, Lars Weinehall

**Affiliations:** 1 Institute for Preventive Medicine and Public Health, Hanoi Medical University, Hanoi, Vietnam; 2 Center for Health System Research, Hanoi Medical University, Hanoi, Vietnam; 3 Epidemiology and Global Health, Department of Public Health and Clinical Medicine, Umeå University, Umeå, Sweden

**Keywords:** climate change, storm, flood, health problems, health system, Vietnam, disasters, disease outbreaks, emergency medical services/utilization, public health

## Abstract

**Background:**

As a tropical depression in the East Sea, Vietnam is greatly affected by climate change and natural disasters. Knowledge of the current capacity of the primary healthcare system in Vietnam to respond to health issues associated with storms and floods is very important for policy making in the country. However, there has been little scientific research in this area.

**Objective:**

This research was to assess primary healthcare system capacities in a rural district in central Vietnam to respond to such health issues.

**Design:**

This was a cross-sectional descriptive study using quantitative and qualitative approaches. Quantitative methods used self-administered questionnaires. Qualitative methods (in-depth interviews and focus groups discussions) were used to broaden understanding of the quantitative material and to get additional information on actions taken.

**Results:**

1) Service delivery: Medical emergency services, especially surgical operations and referral systems, were not always available during the storm and flood seasons. 2) Governance: District emergency plans focus largely on disaster response rather than prevention. The plans did not clearly define the role of primary healthcare and had no clear information on the coordination mechanism among different sectors and organizations. 3) Financing: The budget for prevention and control of flood and storm activities was limited and had no specific items for healthcare activities. Only a little additional funding was available, but the procedures to get this funding were usually time-consuming. 4) Human resources: Medical rescue teams were established, but there were no epidemiologists or environmental health specialists to take care of epidemiological issues. Training on prevention and control of climate change and disaster-related health issues did not meet actual needs. 5) Information and research: Data that can be used for planning and management (including population and epidemiological data) were largely lacking. The district lacked a disease early-warning system. 6) Medical products and technology: Emergency treatment protocols were not available in every studied health facility.

**Conclusions:**

The primary care system capacity in rural Vietnam is inadequate for responding to storm and flood-related health problems in terms of preventive and treatment healthcare. Developing clear facility preparedness plans, which detail standard operating procedures during floods and identify specific job descriptions, would strengthen responses to future floods. Health facilities should have contingency funds available for emergency response in the event of storms and floods. Health facilities should ensure that standard protocols exist in order to improve responses in the event of floods. Introduction of a computerized health information system would accelerate information and data processing. National and local policies need to be strengthened and developed in a way that transfers into action in local rural communities.

Climate change contributes to a rise in the frequency and severity of natural disasters, especially storms and floods that can lead to a number of societal risks and health consequences (1–3). Health effects of climate extremes can be direct, such as drowning and injuries, or indirect and delayed, such as waterborne infections, acute or chronic effects of exposure to chemical pollutants released into flood waters, vector-borne diseases, mental health consequences, and food shortages (4–9). Storms and floods can also disrupt the capacity of healthcare systems to respond to health crises, and affect the overall quality of healthcare (10).

Vietnam is one of the most disaster-prone countries in the world. As a tropical depression in the East Sea, Vietnam is significantly affected by climate change and natural disasters. Over the past 20 years, natural disasters resulted in the loss of over 13,000 lives, and annual damage equivalent to an average 1% of the gross domestic product (GDP) (11). The most damaging and frequent disasters affecting Vietnam are tropical storms and floods. In 2007, an estimated 400 people died as the direct result of storms and floods. The economic loss to society was estimated around VND 11.5 billion (approximately USD 650 million) (12). The impact of climate change and associated events in Vietnam was projected to be serious and an imminent threat to poverty reduction, as well as the achievements of the Millennium Development Goals, which include health goals (13). Among other actions formulated to deal with problems associated with climate change and disasters, the Vietnamese government approved policies such as the 2007 National Strategy for Natural Disaster Prevention, Response and Mitigation to 2020, and the 2008 National Target Program in Response to Climate Change and is developing Law on Climate Change and Law on Disaster Management policies. The key objective of these polices is to establish a feasible action plan to deal effectively with climate change and disaster problems, including storm and flood-related issues (13).

The Vietnamese health system organizational structure consists of four levels that parallel the state administration system—central, provincial, district, and commune. At the central level, the Ministry of Health is the government agency that carries out the state management of healthcare protection and promotion, including preventive medicine, curative care, rehabilitation, traditional medicine, prophylactic and treatment drugs, cosmetics, food safety and hygiene, oversight of medical equipment, and management of public services under ministry control. At the provincial level, the provincial health department is a professional agency managed by the Provincial People's Committee, and works to advise the Provincial People's Committee on state management of local healthcare protection and promotion. The provincial health department performs tasks and duties as authorized by the Provincial People's Committee and legal regulations. The Provincial People's Committee controls the direction, organizational management, payroll, and operations of the provincial health department. The provincial health department is also under Ministry of Health control of technical directions, guidance, monitoring, and inspections. At the district level, the district health bureau is a professional agency under management of the District People's Committee that works to advise the District People's Committee on state management of local healthcare protection and promotion, and performs designated tasks and obligations as authorized by the District People's Committee and provincial health department. The District People's Committee controls the district health bureau in terms of direction, organizational management, payroll, and operations. District health bureau is also under provincial health department control of technical directions, guidance, monitoring, and inspections. The district level also has district hospitals (including polyclinics) and district centers for preventive medicine. The district centers for preventive medicine recently split from district health centers and are under provincial health department stewardship and management. At the commune level, the commune health center is the first formal point of healthcare contact with the government healthcare system. The commune health center provides primary healthcare services, conducts early detection of epidemics, provides care for common diseases and deliveries, mobilizes people to use birth control, teaches preventive hygiene practices, and manages health promotion. The commune health center is responsible to the district health bureau and the Commune People's Committee for local healthcare protection and promotion, and receives technical guidance from the district hospitals. The commune health center also supervises village health workers who are active close to homes and worksites. Every village has a village health worker with 3–9 months of training (14).

The Vietnamese healthcare system is assigned primary responsibility for prevention and response to climate change and disaster-related health issues. The primary healthcare system (including district and commune levels) is the first site of contact between individuals, the family, and community with the national health system. Primary healthcare brings health care as close to where people live and work as possible, and constitutes the first element of a continuing healthcare process. The primary healthcare system is expected to be the frontline for dealing with climate change and natural disaster-related health issues, particularly in rural settings (15).

Extreme weather and climate events interact with exposed and vulnerable human and natural systems and can lead to disasters (3). The concept of adaptive capacity has existed for decades (16–18). However, the most recent definition adopted by the Intergovernmental Panel on Climate Change is ‘the ability of a system to adjust to climate change (including climate variability and extremes) to moderate potential damages, to take advantage of opportunities, or to cope with the consequences’ (19, 20). Current conceptual underpinnings of adaptive capacity are most closely associated with the Intergovernmental Panel on Climate Change characterization of adaptation as an ‘adjustment in natural or human systems in response to actual or expected climatic stimuli or their effects’. Successful adaptation should result in an equal or improved situation when compared with the initial condition, while less successful responses (such as coping) will allow for short-term recovery but continued vulnerability. System coping capacity, or capacity of response is also called adaptive capacity (21, 22). The Intergovernmental Panel on Climate Change distinguishes coping capacity or response from adaptive capacity, and considers both as components of system resilience. Adaptations are referred to as system restructuring after response (21). Some authors apply ‘‘coping ability’’ to short-term capacity or the ability to just survive, and employ ‘‘adaptive capacity’’ for long-term or more sustainable adjustments (23). In general, response capacity is the system's ability to adjust to a disturbance, moderates potential damage, takes advantage of opportunities, and copes with the consequences of the occurring transformation. Capacity of response is a system attribute that existed prior to the perturbation. Broadly speaking, adaptive capacity denotes the ability of a system to adjust, modify, or change its characteristics or actions to moderate potential damage, take advantage of opportunities, or cope with the consequences of shock or stress (24).

Knowledge of the current capacity of the primary healthcare system in Vietnam to respond to health issues associated with storms and floods is important for national policy making. However, there has been little scientific research in this area. The objective of this research is to assess capacity of the primary healthcare system in a rural district in central Vietnam to respond to health issues associated with storms and floods. The key research question was ‘How capable is the primary healthcare system in rural Vietnam to respond to health issues associated with storms and floods, in terms of six system building blocks?’. These included: 1) service delivery, 2) policy/governance, 3) healthcare financing, 4) human resources, 5) information and research, and 6) medical products and technology. Research results are expected to be used by relevant stakeholders and policymakers in Vietnam to bridge national policies with local context and capacities during planning, management, and decision-making.

## Methods

### Study design

This was a cross-sectional, descriptive research study that used quantitative and qualitative approaches. Quantitative methods used self-administered questionnaires. The questionnaires allowed respondents to answer *very good, good, fair, bad, or very bad*. Qualitative methods (in-depth interviews and focus groups discussions) were used to broaden understanding of the quantitative material and to acquire additional information on actions taken. In-depth interviews collected data from healthcare staff at health facilities on different levels. We sought information from the perspective of the health service providers. Focus group discussions were used to expand information from a broader group of informants. Focus group discussions were organized for people representing different parts of civil society, such as the Women's Association, Veterans Association, Farmers Association, Youth Union, and Police. At the commune level, these individuals were local representatives identified through the Commune People's Committee.

### Study scope

We assessed capacity of the study area primary care system (district and commune health organizations) to respond to storm and flood-related health consequences, based on the World Health Organization (WHO) model of six primary care system building blocks: 1) service delivery, 2) policy/governance, 3) healthcare financing, 4) human resources, 5) information and research, and 6) medical products and technology (25).

### Study area

PhuVang district in Thua Thien-Hue province, located in the North Central Coast region of Vietnam, was selected for this study. PhuVang is a rural district that covers an area of 280 km^2^. As of 2010, the total PhuVang population was 171,363. The district was selected because it is a location where storms and floods frequently occur. In 2012, the PhuVang district healthcare organization included PhuVang district hospital with more than 80 beds, PhuVang district health center, PhuVang District Health Bureau, two inter-communal polyclinics, 20 commune health centers, and a network of village health workers.

### Study sample

Health facilities and staff at the district and commune levels from the PhuVang district primary healthcare system were studied. As the provincial health system was responsible for managing and supervising primary healthcare facility activities, interviews were also done with representatives from the Provincial Department of Health and centers for preventive medicine. Focus group discussions were done with local representatives from other sectors and community organizations. The study sample is presented in Table 1.

**Table 1:** The study sample

	Province		District			Commune			
	
	Provincial department of health	Center for preventive medicine	Center for preventive medicine	Hospital	Health Bureau	Health center^a^	Health staff	Non-health sector^b^	Total
Self-administered questionnaires	–	–	1	1	–	20	–	–	22
In-depth interviews	1	1	1	1	1	5	–	–	10
Focus group discussions	–	–	–	–	–	–	5	5	10

a PhuVang District has 20 health centers.

b Representatives from the Committee for Flood and Storm Control, Agriculture and Rural Development, Hydrometeorology Unit, TV/radio station, community organizations (including the Women's Association, Veterans Association, Farmers Association, Youth Union), and local people.

### Research tools

Research tools were a self-administered questionnaire, guidelines for in-depth interviews, and guidelines for focus group discussions. They were developed by a team of experienced researchers from the fields of medicine, epidemiology, and social medicine. The tools were pilot-tested and calibrated before official use.

### Data collection

The study was conducted between January and April 2013. Data collection was conducted by the research team, and consisted of professionals with experience in public health and health systems. The research team visited selected health facilities in the study area to collect the necessary data.

### Data management and analyses

EpiData 3.1 and Stata10 were used to enter and analyze quantitative data. Analysis of the qualitative data was inspired by descriptive content analysis techniques, which focus on the manifest content (i.e. look at the most obvious and straightforward meanings of a text) (26). Data were organized into six themes, corresponding to the six WHO building blocks listed above (25).

### Ethical considerations

Permission to conduct this study was approved by Hanoi Medical University and Thua Thien-Hue provincial health authorities. Informed consent was obtained from each informant.

## Results

### Service delivery

#### Prevention activities

For the prevention activities, respondents were asked to list prevention activities implemented for storm and flood-related health problems at the local health facilities and how the health facilities respond to storm and flood-related health problems. The quantitative survey showed that different population-based health promotion activities intended to improve local knowledge about storm and flood-related health problems was regularly implemented before, during, and after the storm and flood seasons. The main health promotion activity topics were about health risks associated with storms and floods, prevention and first-aid solutions, water, sanitation, nutrition issues, and disinfectant techniques after hazardous events. These prevention activities were implemented by the district health center, the commune health center, and the village health worker network through community meetings and public loudspeaker announcements. Respondents from 18 of 20 of the studied facilities (90%) rated their prevention activities as fair or good. Only 2 of 20 (10%) reported that they performed their prevention tasks poorly. The main difficulties encountered while implementing prevention activities were a lack of staff and funding. Laboratory equipment for disease surveillance and outbreak confirmation was insufficient. In addition, water and sanitation systems were poor, which made the prevention work more difficult. Findings from focus group discussion also showed that the preventive medicine team efforts were appreciated by the community. Focus group discussions showed that preventive medicine services (counseling on prevention and first-aid for storm and flood-related health problems, vaccinations against six preventable diseases, including diphtheria, tetanus, pertussis, poliomyelitis, measles and tuberculosis) were accessible to the local population and brought health benefits to the community.

#### Treatment activities

For treatment activities, respondents were asked to list treatment activities used for storm and flood-related health problems at the health facilities, rate the treatment activities, the number of patients the health facility treated during the past 5 years, and advantages and disadvantages of treatments for storm and flood-related health problems at the health facility. Quantitative and qualitative data revealed that emergency plans were established before storm and flood seasons at district and commune health facilities. At district hospitals, ambulance and emergency services were prepared. At the commune health station, first-aid services were readily available. Village health worker networks were provided with some medicines, supplies, and basic medical equipment needed for first-aid. All the interviewees reported that they were confident and happy with the quality of the first-aid services because standardized first-aid kits and protocols were available. All of the health staff had a chance to be involved in disaster simulation exercises to test response mechanisms. Most (79.9%) considered the treatments for common illnesses (e.g. dermatitis, skin infections, fungal infections, conjunctivitis, digestive problems, diarrhea) as fair or good. However, most (72.9%) raised concerns about the availability and quality of surgical services provided at the district hospital (by regulation, surgical services were not provided at commune health centers). Thirty-six percent of study respondents reported that treatment services (especially surgical operations) were sometimes not available during storm and flood seasons. Sixty-three percent rated the emergency referral system as poor. The same opinions on availability and quality of common services, especially of surgical operation services and emergency referral systems, were also identified in the focus group discussions with representatives from other sectors, community organizations, and local people. Building damages, electrical outages, and inadequacy of professional staff were reported as the main causes for primary healthcare system dysfunction during storm and flood seasons. Table 2 presents quantitative survey results of selected diseases or illnesses reported by the commune health centers before, during, and after the 2012 storm and flood season.

**Table 2:** Selected disease and illness cases reported by the CHC before, during, and after the 2012 storm and flood season, presented as average number of cases per month per center

Selected disease	Before (July–August 2012)	During (September–October 2012)	After (November–December 2012)
Injury	1.3	3.8	2.1
Conjunctivitis	1.6	1.4	3.4
Skin diseases (pruritus)	2.1	2.2	7.3
Gastrointestinal diseases (diarrhea, cholera, dysentery)	2.3	3.5	7.8

### Policy and governance

Respondents were asked about the availability of instructive documents that requested participation in prevention activities for storm and flood-related health problems, the frequency and timeliness in which the health facilities received those documents, and advantages and disadvantages to implementation of the instruction and guidance documents for prevention activities. Similar questions were asked about treatment activities.

While the Law on Climate Change and the Law on Disaster Management Policies were being developed, the key documents underpinning disaster risk reduction policies and strategies were the Ordinance on Prevention and Control of Floods and Storms (adopted by the Standing Committee of the 9th National Assembly of the Socialist Republic of Vietnam in 1993), the 2007 National Strategy for Natural Disaster Prevention, Response and Mitigation to 2020, and the 2008 National Target Program in response to Climate Change. According to these policies, the Ministry of Health is a member of the Central Steering Committee for Flood and Storm Control (chaired by the Ministry of Agriculture and Rural Development) and has the main responsibility in prevention and dealing with climate change-related health issues.

At the provincial, district, and commune levels, committees for Prevention and Control of Floods and Storms (with the health sector as a member) are directed by the People's Committee of the same level. Each year, each involved sector (including the health sector) develops a plan for prevention, control, and response to consequences of floods and storms.

At the local level, district emergency plans for storms and floods (responsibility of the district Agriculture and Rural Development Bureau) were reviewed and updated annually. However, the plans focused largely on disaster response rather than prevention. The plans lacked clear information on the coordination mechanism among different participating sectors and organizations. Plans did not clearly define the role of primary healthcare in implementation of the health emergency plan, and did not generally address the needs of vulnerable groups or gender considerations. Budgets for health emergency plans were also missing. Every facility reported that they lacked an emergency plan and that they were passive in responding to disaster problems. Health facilities did not have specific job descriptions for handling storm and flood-related health problems within each organization.

Most respondents (84.3%) reported that they knew of some general policy documents on prevention, control, and response to floods and storms. However, they did not know of any policy pertaining to roles and specific tasks of the primary healthcare system to respond to climate change and disaster-related health issues. Sixty-eight percent stated that legal and policy framework to support primary healthcare system response to storm and flood-related health problems have been inadequate.

Social mobilizations for responding to storm and flood-related health problems were good. Apart from health sector efforts, a number of climate change and disaster management projects funded by international donors or non-governmental organizations (e.g. The Red Cross, CARE, and ADB) had been implemented. The projects made substantial contributions to improving public awareness on climate change, disasters, and associated issues including health problems. Other sectors such as agriculture and rural development, the hydrometeorology unit, the radio station, and community organizations were also active in health promotion events and disseminating health promotion messages to local communities. All of the respondents said that to be more effective, better intersectoral coordination was needed.

### Healthcare finance

Healthcare finance findings were extracted from questionnaires and in-depth interviews. Questions were asked about availability of separate funding sources for storm and flood-related health problem prevention and treatment activities. These included questions on how to allocate funds for these activities, special mechanisms for patients derived from the health sector, sufficiency of funding for annual operations, availability of financial support from localities, how this financial support affected local residents and health facilities, and advantages and disadvantages of the financial policies at the health facility as related to implementation of prevention and treatment activities relating to storms and floods.

At the local level, activities of the Committees for Prevention and Control of Floods and Storms were financed from the National Reserve budget, state contingency budget, and local reserve funds for prevention and control of storms and floods. Local citizens aged 18–60 years and companies or agencies located in the community are mandated to make financial contributions to the local reserve fund. However, the budget amounts for prevention and control of storm and flood activities were limited and had no specific items for healthcare activities.

Except for the PhuVang District Health Bureau, the agency belonged to and was funded by the District People's Committee. PhuVang district and the commune health organizations received funding from the Thua Thien-Hue Provincial Health Bureau (a government budget) to pay staff salaries and other recurrent expenditures (i.e. electricity, water, meetings, travel). Despite playing important roles in health promotion activities and emergency care during storm and flood seasons, village health workers received little remuneration from the district health center (USD10–20 per person per month).

The health facilities reported that they received no additional budget from the health system for prevention and treatment of climate change and disaster-related health issues. In the event of a natural disaster, the PhuVang district health center had to seek financial support from the District People's Committee and/or the Thua Thien-Hue Provincial Health Bureau. Commune health center could also ask for support from the Commune People's Committee. In-depth interviews with the representatives from health facilities revealed that these extra amounts of money were normally small and used to cover small health staff allowances incurred while providing emergency services (e.g. instant food for patients, disinfection chemicals). Procedures to acquire this financial support were typically time-consuming.

The PhuVang district received some in-kind support from Thua Thien-Hue Pharmaceutical Company (i.e. medicine, medical equipment). The district also had projects on climate change and disaster management that were funded by international donors. Such funding was used to deploy specific activities such as capacity building, development of early-warning systems, or purchasing equipment. Again, the international funding allocated for implementing primary healthcare services was very limited.

In PhuVang district, 2011 health insurance coverage was nearly 70%. Funding from the health insurance fund was used to finance almost all health services for storm and flood-related health problems.

### Human resources

Human resources results were drawn from questionnaires and in-depth interviews. Demographic information on health staff was collected. The primary questions were about the number staff and the frequency of participation in training courses on prevention and treatment activities on storm and flood-related health problems during the past 5 years, the skills and knowledge needed to implement those activities effectively, assessment of the quantity and quality of the health workforce at their health facility, and opinions on the advantages and disadvantages in implementation of these prevention and treatment activities.

As of 2012, the PhuVang healthcare system had 258 employees: 147 worked at the district health level (102 in the district hospital, 41 in the district health center, four in the district health bureau), and 111 worked in the 20 commune health centers. The district hospital had two doctors with specialization level 1 on emergency care, and four doctors with specialization level 1 on surgical operations (with one of the four specialized in injury and trauma). The district health center had one master of public health and seven doctors of specialization level 1 in preventive medicine and public health. Each of the 20 commune health centers had at least one medical doctor. The number of commune health center staff was 3.4 per 1,000 populations. In addition, there were 369 village health workers supporting commune health center's health activities. The PhuVang healthcare system did not have any environmental health specialists.

During the last 3 years, all PhuVang professional healthcare staff received at least one training session on a topic relevant to prevention and control of climate change and disaster-related health issues. The training sessions included topics such as underwater rescue, first-aid, and transporting victims. Preventive medicine staff took part in the training on raising public awareness of the hazardous impacts of storms and floods, and communicable disease surveillance and control. Clinical staff attended training on emergency care, diagnosis, management, and treatment of injuries of injury, drowning, and snake bites. However, the quantity and topics covered by the training sessions were inadequate. Eighty-six percent of respondents reported that the number of training sessions did not meet actual needs. Seventy-nine percent stated that they needed training on specialized skills such as disaster planning, development and management of emergency plans, and effective referral of patients during storm and flood seasons.

### Medical products and technology

Medical product and technology was assessed with questions that asked for a list of a basic unit of medicine, medical devices, medical equipment for mobile emergency teams, availability of related medicines, availability of medical equipment, availability of treatment protocols, and advantages and disadvantages of these medicines and medical equipment for storm and flood-related health problems.

Lists of essential medicines and medical equipment were well described in the 2012 Thua Thien-Hue Provincial Health Bureau plan for storm and flood prevention and control. These essential medicines and medical equipment for emergencies were available in all of the facilities. Seventy-eight percent of respondents reported that their health facilities always had sufficient essential medicines for treatment of storm and flood-related health issues. Twenty-two percent reported that their facilities experienced temporary shortages of some medicines and supplies during some storm and flood days. First-aid kits were available at each health facility. Ninety-three percent of participants thought they had enough medical equipment for first-aid services during storms and floods. Only 7% thought that they needed more kits to distribute to each of the district village health workers.

The health facilities reported that they had protocols for drowning rescue, electrical shock, and first-aid for injuries, but our field visits revealed that only half (three out of six) had such protocols. None of the six facilities had a specific emergency plan.

### Health information system

The results for health information systems were extracted from the questionnaires. Health staff were asked how they stored information and reports on prevention and treatment activities, how they informed local inhabitants, whether there was a separate report on prevention and treatment activities for storm and flood-related health problems, how they cooperated with the meteorological agency, and advantages and disadvantages of data management and reporting.

Consistent with the health sector reporting system of Vietnam, the PhuVang commune health centers sent annual reports to the district health center. The district health center and district hospital submitted annual reports to the Thua Thien-Hue Provincial Health Bureau. The Thua Thien-Hue Provincial Health Bureau then sent provincial annual reports to the Ministry of Health.

During storm and flood seasons, the commune health center submitted daily health reports to the district health center and Commune People's Committee. The district health center subsequently made an overall district report for submission to the provincial health department and District People's Committee. These reports were in paper formats and not stored well. Other vertical programs also required the commune health centers and district health center to complete several reports and forms. At the district hospital, patient records were not organized in a way that facilitated patient management. System information on patient referrals and back-referrals were usually missing. The qualitative study found that data that could be used for planning and management (including population and epidemiological data) were largely lacking.

A vulnerability assessment, a method to identify hazards and determine their possible effects on a community, activity or organization, was not done in the PhuVang district. An early-warning system was not officially developed, and local residents were dependent on disaster announcements through mass media and a community loud speaker system.

## Discussion

This section discusses six building blocks of primary health system in PhuVang district, Thua Thien-Hue province, in terms of responding to storm and flood-related health problems. Functional characteristics of a primary health system, including availability, affordability, accessibility, and quality according to WHO recommendation are addressed.

### Service delivery

Availability of health services is an important aspect of a well-functioning primary healthcare system (27, 28). Disease surveillance and outbreak confirmation activities that support preventive medicine services were not well-implemented in the PhuVang district. Population-based health promotion campaigns about storms and flood-related issues had been regularly implemented. As recommended by the WHO, close disease surveillance systems should be in place and functional. Surveillance acts as an early-warning system for infectious disease outbreaks, provides information for identifying known and previously unknown non-infectious health hazards, and ensures that health services address the needs of the population and vulnerable groups (29).

In line with previous studies (1, 2, 4–10), our study showed that there was a relationship between storms and floods and the number of disease or illness cases (including injuries) in the study area. During storm and flood events, accidents or emergencies were the most common reasons for people to seek health care. However, medical emergency services, especially surgical operations and referral systems, were not always available during storm and flood seasons due to building damage, electricity outages, or inadequate professional staff. Similar situations were found in rural Ethiopia in 2007 (30) and India in 2008 (31). According to the WHO, timely and effective emergency services could save many lives during disaster events, and health facilities should prepare for changes in the populations who attend outpatient clinics because floods might cause population displacement (32). Therefore, dysfunction of emergency services in the primary health system should be actively prevented. Existing guidelines for dealing with disaster-related health problems such as the WHO emergency response framework (32) and the SPHERE project standards of care in mass casualty events (33) should be referenced. Alternate sources for electricity, emergency transportation, and additional health professional availability are necessary during storm and flood seasons (28, 34).

### Policy and governance

National policies for responding to storm and flood-related health problems were available but needed to be strengthened and developed in order to transfer into action in local rural communities. Our study showed that district emergency plans were available. However, these plans focused largely on disaster response rather than prevention. The plans did not generally address the needs of vulnerable groups or have gender considerations. The plans did not clearly define the role of primary healthcare and had no clear information on coordination among different sectors and organizations. As a result, they did not have a clear working plan to prepare for or prevent climate hazard consequences. They were passive in responding to disaster problems. For example, to implement one prevention activity such as chemical spraying, health staff had to approach households and public areas with other stakeholders. However, those representatives lacked enthusiasm, which sometimes resulted in delayed or ineffective chemical spraying. In other words, there needs to be a working mechanism among participating stakeholders to effectively implement treatment and prevention activities. Because there was no clear cooperation with stakeholders, and passivity, the health staff did not have specific job descriptions within their organizations for handling storm and flood-related health problems.

Similar situations were reported in Mozambique, where emergency response plans for storm and flood-related health problems were not clearly described (35). A study from rural India reported a lack of clear coordination between the state and district levels, and between district and block and health center levels (31). Successful emergency plans depend on the collaboration of multiple agencies. Emergency plans for health care should have a clear role in different sectors, as well as multisectoral coordination (36–42). Ideally, emergency response should be led and coordinated by a body at the central and local levels and include all relevant health sector disciplines to address all potential health risks. More effort is needed for specific consideration of gender and vulnerable groups such as children, women, and the elderly (43).

### Healthcare financing

An important aspect of a good financial mechanism for addressing storm and flood problems is to prepare for surge capacity and stockpile resources (34). To avoid storm and flood-related health problems, a good health financing system is also needed to ensure better access to services needed by patients, and the safety of health facilities and equipment (44). The PhuVang district budget for prevention and control of storm and flood-related activities was limited and lacked specific items for healthcare activities. Little additional funding was available, and the procedures to procure the funding were time-consuming. A study from Mozambique found that the governmental budget for emergency response was critical, but procedures to access funds needed simplification to improve financial response (45). Another study from India also reported that existing resources were inadequate during flood events (31). Funding for flood response should be calculated on past experiences, and each facility should be granted emergency contingency funds in their annual budget (31, 46).

### Human resources

Human resources are a key challenge during disasters, and the problem is more pronounced in rural settings (47). Medical rescue teams were established in PhuVang district, but there were no epidemiologists or environmental health specialists to address epidemiological issues. For continuity of essential services and to minimize risks of potential communicable disease outbreaks through prevention, additional health staff with appropriate knowledge, competencies, and skills are important to provide support to ‘fatigued’ health officers. In 2012, the PhuVang district population in Thua Thien-Hue province was 178,103 persons (48). If health staff totaled 258 persons, the ratio of health staff to population would be 14/10,000. This ratio is significantly lower than the 23/10,000 persons suggested by WHO as necessary to reach related millennium development goals (MDGs) [fewer than 23/10,000 generally fails to achieve adequate coverage rates for selected primary healthcare intervention (49)].

Training staff in management of mass casualty incidents holds the key to effective and optimum use of available resources (50). However, in the PhuVang district, training on prevention and control of climate change and disaster-related health issues did not meet actual needs. Health staff skills relevant to storm and flood-related health problems were inadequate. Similar results were found in rural India (31) and Mozambique (50). Primary healthcare staff need to be provided with training topics such as dealing with floods, analysis of flood effects, scenario planning based on information from situation management (from the worst to the best), and monitoring of disease situations (51). A comprehensive training and education needs’ assessment is important for identification of the skills required for performance of specific health-related tasks in crisis preparedness and response.

### Medical products and technology

As stated in the 1993 Ordinance on Prevention and Control of Floods and Storms, the health sector is responsible for building reserves of medication and medical equipment, instructing and disseminating the use of emergency techniques, and prevention techniques for epidemics and diseases likely to occur after storms and floods. Healthcare organizations at all levels have a responsibility to fulfill this regulation.

Health facilities have to access standard treatment protocols to ensure uniformity of treatment for populations affected by storms and floods. However, our study revealed that emergency treatment protocols were not available in all health facilities. In the absence of Ministry of Health-guided rescue protocols, WHO treatment protocols or Medicine Sans Frontiers guidelines could be used (31).

The WHO recommends the Interagency Emergency Health Kit (containing essential medicines and medical devices for primary healthcare workers with limited training) which contains oral and topical medicines to be used and tailored to reflect local availability of medicines, devices, and common flood types (27, 52). Reviewing the inventory list of stocked medicines and equipment, together with the essential medicine and device list, would show whether supplies match disaster response needs, as well as the location and accessibility of these supplies (53). More kits might need to be distributed to each village health worker in the district.

### Health information system

An important issue for effective disaster response was having the information to make a disaster response plan. A plan for a quick response includes the medical relief activities, requires access to health services data, and facilities during the pre-disaster phase. In the PhuVang district, data useful for planning and management (including population and epidemiological data) were largely lacking. A study from Indonesia showed that lack of necessary information or a prolonged deficiency of information meant that aid agencies were unable to efficiently distribute relief and provide assistance (54). Another study from Mozambique showed a similar situation (55). Introduction of a computerized health information system in Uganda resulted in health workers putting greater value on generated data, and accurate access to information was extremely important (56).

The PhuVang district lacked a disease early-warning system (DEWS) based on epidemic surveillance. DEWS plays an important role in overseeing the risks and signs needed for a timely response to future flood disasters (57). It remains a big challenge to assure that everyone receives timely warnings, understands the warnings, and can potentially take prompt action (55). Pakistan experienced an extreme flooding in 2010 that affected approximately 18 million persons. In response to the emergency, the Pakistan Ministry of Health and WHO enhanced existing DEWS for outbreak detection and response. Those improvements in DEWS increased system usefulness in subsequent emergencies. An effective community-based early-warning and evacuation system, including cyclone shelters for evacuation, was a crucial factor in saving many lives (58).

### Limitations of the study

Epidemiological studies of the impact of storms and floods, identifying risk groups, and delays in understanding the full extent of disease outcomes (acute and chronic) need further investigation. This study was not able to include such studies, but they are necessary to achieve the full potential of secondary or tertiary prevention strategies in the healthcare sector.

## Conclusion

Primary care system capacity in rural Vietnam is inadequate for responding to preventive and treatment healthcare for storm and flood-related health problems. Developing clear facility preparedness plans with detailed standard operating procedures during floods and identifying specific job descriptions will strengthen the future response to floods. Each facility should have contingency funds available for emergency response. Health facilities should ensure that Ministry of Health emergency treatment protocols for healthcare delivery are available. Introduction of a computerized health information system resulted in health workers putting greater value on generated data and should be used to speed up information and data processing. National and local policies need to be strengthened and developed so that they can be translated into action in rural communities.
